# Protein Kinase C: An Attractive Target for Cancer Therapy

**DOI:** 10.3390/cancers3010531

**Published:** 2011-02-01

**Authors:** Barbara Marengo, Chiara De Ciucis, Roberta Ricciarelli, Maria A. Pronzato, Umberto M. Marinari, Cinzia Domenicotti

**Affiliations:** 1 G. Gaslini Institute, L.go G. Gaslini 5, 16147, Genoa, Italy; E-Mail: bmare2002@yahoo.it; 2 Department of Experimental Medicine, General Pathology Section, University of Genoa, Via L.B. Alberti 2, 16132, Genoa, Italy; E-Mails: cdeciucis@yahoo.it (C.D.C.); maidep@unige.it (M.A.P.); ricciarelli@medicina.unige.it (R.R.); umm@unige.it (U.M.M.)

**Keywords:** apoptosis, PKC, cancer, chemoresistance

## Abstract

Apoptosis plays an important role during all stages of carcinogenesis and the development of chemoresistance in tumor cells may be due to their selective defects in the intracellular signaling proteins, central to apoptotic pathways. Consequently, many studies have focused on rendering the chemotherapy more effective in order to prevent chemoresistance and pre-clinical and clinical data has suggested that protein kinase C (PKC) may represent an attractive target for cancer therapy. Therefore, a complete understanding of how PKC regulates apoptosis and chemoresistance may lead to obtaining a PKC-based therapy that is able to reduce drug dosages and to prevent the development of chemoresistance.

## 1. The PKC Family: Its Structure and Activation

Protein kinase C (PKC) was originally discovered by Yasutomi Nishizuka in 1977 as a histone protein kinase activated by calcium and diacylglycerol (DAG), phospholipids and/or phorbol esters [1]. It is known that the PKC family consists of serine/threonine-specific protein kinases that differ in their structure, cofactor requirement and substrate specificity [2]. Due to biochemical properties and sequence homologies, PKCs are divided into three subfamilies: firstly, classical or conventional PKCs (cPKCs; PKCα, PKCβI, PKCβII and PKCγ), which are calcium dependent and activated by both phosphatidylserine (PS) and DAG. Secondly, novel PKCs (nPKCs; PKCδ, PKCε, PKCη and PKCθ), which are calcium independent and regulated by DAG and PS, and finally, atypical PKCs (aPKCs; PKCζ and PKCλ), which are calcium-independent and do not require DAG for activation, although PS can regulate their activity [3-5].

**Figure 1.**
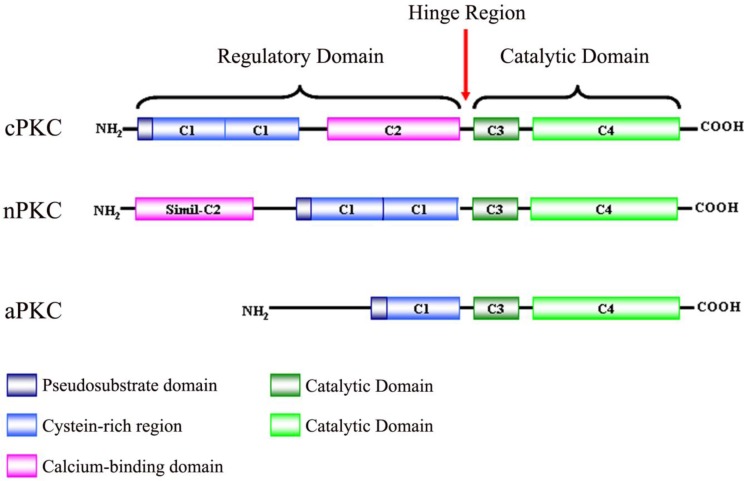
Schematic representation of protein kinase C (PKC) isoenzyme structure and classification. The PKC family is divided into three subfamilies: classical (cPKCs; PKCα, PKCβI, PKCβII and PKCγ), novel PKCs (nPKCs; PKCδ, PKCε, PKCη and PKCθ) and atypical PKCs (aPKCs; PKCζ and PKCλ). PKC has four conserved domains (C1–4): C1 has cysteine-rich motifs that form the diacylgylcerol (DAG) and phorbol ester binding site; C2 contains the recognition site for acidic lipids and calcium binding site; C3 and C4 form the ATP and substrate binding sites.

PKC isoenzymes share the same structural properties (Figure 1), namely, a carboxyl-terminal kinase domain linked by a flexible hinge-segment to an amino-terminal region containing regulatory modules [6,7]. These regulatory modules confer sensitivity to the DAG (C1 domain) or Ca^2+^ (C2 domain), although some isoenzymes have variants of these modules that do not bind ligands [8]. cPKCs contain both C1 and C2 domains, C1 that binds DAG and phosphatidylserine, C2 that binds anionic lipids in a Ca^2+^-dependent manner [9]. The C1 domain, with cysteine-rich zinc finger structures, is also the binding site for the tumor-promoting phorbol esters [10] which competitively bind with DAG [11]. nPKCs contain tandem C1 domains that bind DAG and a variant of the C2 domain that is unable to link Ca^2+^ and, as a result, these isoenzymes are not sensitive to Ca^2+^, but their affinity for DAG is two orders of magnitude higher than that for the cPKCs [12]. aPKCs contain a variant of the C1 domain that binds PIP3 or ceramide (not DAG or PMA) and a protein-protein interaction PB1 (Phox and Bem 1) domain that mediates interactions with other PB1-containing scaffolding proteins including p62, partitioning defective-6 (PAR-6) and mitogen-activated protein kinase (MAPK) modules like MEK5 [13,14].

All isoenzymes have a conserved carboxyl-terminal tail (CT) that serves as a phosphorylation-dependent docking site for key regulatory molecules and an autoinhibitory pseudosubstrate sequence that maintains PKC in an inactive state.

Situated between the regulatory domain and the catalytic domain, is the V3 region, which is accessible to proteolytic cleavage upon activation and conformational change of PKC [15]. Cleavage at this site leads to the release of a constitutively active catalytic domain, suggesting that many inhibitory intra-molecular interactions occur between the regulatory and catalytic domains.

PKCs may acquire the stability and the catalytic competence by a process of maturation consisting of constitutive phosphorylations [6,16]. Recently, the central role of heat shock protein-90 (HSP90) and of the mammalian target of rapamycin complex 2 (mTORC2) in this maturation process has been demonstrated [17,18]. Once fully processed and phosphorylated, PKCs can respond to second messengers and can also phosphorylate downstream targets. Activated PKCs are subject to down-regulation by ubiquitination and proteasomal degradation after prolonged activation with tumor-promoting phorbol esters, as well as to phosphatase activity [19]. In this regard, it has been demonstrated that the PH domain leucine-rich repeat protein phosphatase (PHLPP) regulates the dephosphorylation step, preceding the downregulation of PKC [20]. This process represents the termination of the life cycle of conventional and novel PKC isoenzymes. In the absence of chronic stimulation, these PKC isoforms have a long half-life whereas sustained activation with phorbol esters results in their rapid degradation [21].

PKCs are subject to a complicated cellular redox regulation. Selective oxidative modification at the N-terminal regulatory domain induces PKC activation [22] while alterations at the C-terminal catalytic domain result in complete inactivation of the kinase [23]. Oxidant treatment of PKC produces a form that does not bind phorbol esters and is catalytically active in the absence of calcium and phospholipids. PKC catalytic domains are inactivated by the loss of free sulphydryl groups required for its function, thus making PKC a potential target for anticancer agents, as well as tumor promoters [24].

Studies *in vivo* and *in vitro* confirm the biphasic behavior of PKC in response to different oxidative damages. High doses of pro-oxidant compounds (carbon tetrachloride, ethanol) cause hepatic PKC inactivation and proteolytic degradation, while low doses induce stimulation of kinase activity [25].

Indeed, Ward *et al.* have suggested that depletion of GSH during oxidative stress removes a mechanism for negative regulation of PKC and, consequently, provides a permissive environment for PKC activity and tumor promotion [26].

Consistent with their different biological functions, PKC isoforms differ, not only in their structure and mode of activation, but also in their tissue distribution, subcellular localization and substrate specificity. The activation of PKC isoenzymes results in changes in their subcellular location following translocation to specific anchoring proteins, collectively named, “receptors for activated C kinases” (RACKs) [27]. RACKs act as molecular scaffolds that are able to localize specific PKCs to distinct membrane microdomains in close proximity with their allosteric activators and unique intracellular substrates. Moreover, it has been discovered that the C2 domain is the region within the regulatory domain of PKC that interacts with RACKs [28,29].

More information about PKC subcellular localization has also been obtained from several studies in which phorbol esters were employed to translocate single PKC isoforms to a specific cellular compartment. In particular, TPA (12-O-tetradecanoyl-phorbol-13-acetate) induced the translocation of PKCα and PKCδ from the cytosol to the plasma membrane and nucleus [30,31] and of PKCε to the Golgi membranes, thereby modulating Golgi functions [32]. However, in the last twenty years, use of green-fluorescent-protein (GFP) fusion proteins have facilitated the monitoring of the PKC's localization in living cells [33,34].

PKC isoenzymes modulate a plethora of biological functions, including cell growth, differentiation, apoptosis, transformation and tumor development [35]. Nevertheless, the way in which PKC isoform specificity *in vivo* covers these processes is not clear, since all cells and/or tissues express more than one PKC isoform that acts in a redundant manner [36]. Due to the signaling amplitude of PKC being ultimately dependent on the levels of PKC poised in the cell, targeting mechanisms that control the levels of PKC offer an alternative approach to controlling PKC signaling.

## 2. PKC Isoforms: Physiological Functions and Role on Cancerogenesis

The tumor promoting properties of phorbol esters have been known for many years and are well documented in animal models of human cancer. The discovery of PKC, as the phorbol ester “receptor”, has led to a heightened interest in the contribution of these kinases to tumorigenesis and tumor progression [37,38].

Overall, the function of PKC in cancer is complex because much of the data indicate that the isoenzymes subtly regulate many pathways involved in cellular transformation [39].

The PKC isoforms most commonly associated with increased proliferation and/or survival, PKCα and ε, are most overexpressed in human cancer and represent potential oncogenes.

PKCα has been associated with several cell functions and its activation with phorbol ester tumor-promoters is associated with the inactivation of E-cadherin, a key factor in the regulation of cell to cell contact, leading to multi-layered cell growth [40]. Furthermore, PKCα modulates membrane re-modeling by stabilizing F-actin and this effect is in opposition to that of PKCε, which induces early actin disruption and basolateral membrane endocytosis [41].

Interesting studies have demonstrated that the α isoenzyme may act as a tumor promoter or as a tumor suppressor [42]. For example, overexpression of PKCα has been demonstrated in tissue samples of prostate, endometrial and high-grade urinary bladder [43,44], up-or down-regulation of PKCα has been observed for hematological malignancies [45] while down-regulation of PKCα has been described in basal cell carcinoma and colon cancers [46,47].

This isoform has been studied extensively in breast cancer cells and contradictory results have been found [48-50]. Recently, it has been reported that PKCα activity supports migration of breast cancer cells *in vitro* and its overexpression correlates to tumor grade, proliferating activity and poor prognosis [51].

In addition, PKCα overexpression is correlated with tumor size and the TNM stage of hepatocellular cancer (HCC) and its levels may be a prognostic marker also in these patients [52]. Consequently, strategies to reduce the expression of this isoenzyme might be useful in cancer therapy. However, since PKCα plays multiple roles in cell physiology and pathology, targeting its downstream signals may be even more beneficial than just targeting the specific isoform. In this regard, it has been demonstrated that the suppression of p38MAPK markedly reduced the invasiveness of human HCC cells [53].

Overexpression of PKCβ can contribute in several ways to tumor formation, being involved in tumor host mechanisms such as inflammation [54] and angiogenesis in breast cancer [55] and in retinal tissue [56]. Elevated expression of PKCβ seems to be an early event in colon cancer development [57] and transgenic overexpression of PKCβII in the intestine induces hyper-proliferation and an invasive phenotype in epithelial cells [58,59]. Consistent with this, the PKCβ specific inhibitor enzastaurin inhibits the activation of the AKT-GSK3 dependent survival pathway in colon cancer cells, as well as in mouse xenograft models [60].

In patients with diffuse large B-cell lymphoma, PKCβ is one of the most overexpressed genes [61] while the loss of PKCβ expression has been observed in melanoma cell lines [62].

PKCγ is mainly expressed in neuronal tissues [63] and there is little information regarding its role in tumor formation. Cell transformation of mammary epithelial cells following PKCγ overexpression has been described, but it is not known whether this contributes to breast cancer formation [64]. Surprisingly, PKCγ has been shown to be a positive prognostic factor for some forms of B-cell lymphomas [65].

PKCδ, a ubiquitously-expressed isoenzyme, is implicated in various cellular processes such as proliferation, differentiation and apoptosis. The diverse effects that PKCδ could exert on cell survival are dependent on its subcellular localization. For example, the δ isoenzyme translocates to the Golgi in response to IFN-γ and ceramide [66], to the nucleus in response to etoposide and irradiation [67] and to the mitochondria in response to UV radiation, phorbol 12-myristate 13-acetate and oxidative stress [68]. In this regard, we have previously shown that glutathione (GSH) depletion induced by L-buthionine-S,R-sulfoximine (BSO) in neuroblastoma cells caused ROS overproduction, PKCδ translocation to the mitochondria and apoptosis [69].

Analyzing the role of PKCδ in cancer progression, PKCδ can act as either a positive or a negative regulator of tumor progression [70]. In this context, it has been demonstrated that the down-regulation of PKCδ with prolonged phorbol-ester treatment of Src-overexpressing fibroblasts confers a malignant phenotype [71], suggesting a tumor-suppressor role for this isoform. On the other hand, it has been found that pro-tumorigenic sonic hedgehog (SHH) signaling and Wnt signaling are dependent on PKCδ/ERK pathways [72].

In relation to specific types of malignancy, PKCδ may be overexpressed in colon cancers and down-regulated in malignant gliomas, bladder carcinomas and endometrial tumors [73,74]. Recently, the expression of PKCδ in human breast cancer has been investigated and an association between elevated PKCδ expression and a poor outcome has been found [75]. Moreover, PKCδ is likely to play a major role in anti-estrogen resistance in breast cancer cells and has been linked with acquired resistance to tamoxifen in breast cancer patients [76].

Conversely, PKCδ activation in prostate cancer serves to promote extrinsic apoptosis through the release of death receptor ligands [77]. Since androgens modulate PKCδ at a transcriptional level, both androgen depletion and androgen receptor RNA interference that suppress the δ isoenzyme triggered apoptosis, suggesting that the hormonal regulation might be a therapeutic approach to modulate PKCδ and its downstream signals [78].

In addition, the overexpression of PKCδ in human cutaneous squamous carcinoma (SCC) cell lines induced apoptosis and suppressed tumorigenicity, making PKCδ a potential tumor suppressor gene for SCCs. In this regard, it has been recently demonstrated that PKCδ gene expression is suppressed in human SCCs, probably via transcription repression [79].

On the contrary, this isoform was overexpressed in human ductal carcinomas and the stable overexpression of this kinase in a human pancreatic carcinoma cell line (PANC1) induced a more malignant phenotype when these cells were inoculated into nude mice [80]. PKCδ has also been linked to an inhibitory role in cell autophagy, suppressing the catabolic process in pancreatic cancer [81].

PKCε is the only isoenzyme that has been considered as an oncogene [82] and the first hint that PKCε may be involved in malignancy came from the study of Baxter *et al.* [83]. In addition, it has been seen that overexpression of PKCε in NIH 3T3 fibroblasts caused increased saturation density, facilitating growth in soft agar and induced tumor formation in nude mice [84].

Similarly, it has been found that the overexpression of this isoform conferred a metastatic phenotype to colonic epithelial cells [85,86]. In addition, PKCε has been shown to be an important mediator of squamous cell carcinogenesis and its overexpression in mouse epidermis caused development of SCC following application of dimethylbenz(a)anthracene and TPA protocol or ultraviolet radiation [87]. The level of PKCε was also increased in primary high-grade astroglial brain tumors [88] and overexpression of dominant-negative PKCε inhibited proliferation of human astrocytoma cells [89]. Moreover, PKCε activation has been linked with invasiveness of human renal cell carcinomas [90] and with aggressive, motile phenotype in breast cancer cells [86] and in human head and neck squamous cell carcinoma [91].

Although, the way in which PKCε modulates cell motility is not completely defined, it has been observed that PKCε promotes actin polymerization [92] and it drives cell motility, in part, through the downstream activation of small Rho GTPases, specifically RhoA and/or RhoC [86], the phosphorylation of Akt [93] and of Stat3 [94].

Overexpression of PKCε has been found in human prostatic tumors and it is associated with the conversion from an androgen-dependent to androgen-independent state [95]. The PKCε gene is also amplified in 28% of thyroid cancers and a chimeric/truncated version of this isoform has been cloned from human thyroid cancer cells [96]. At present, there is no existing literature to document PKCε overexpression in samples from patients with hematopoietic cancers [97].

## 3. PKCs: a Cell “Fulcrum” Able to Modulate Apoptosis and Cell Survival

Apoptosis is a multi-stage process that is vital for the maintenance of homeostasis in multicellular organisms. However, in cancer, the evasion from the programmed cell death plays a role in chemoresistance. In this regard, it has, in fact, been demonstrated that the activation of PKCs can be associated with resistance but can also increase sensitivity to chemotherapy [98]

From this point of view, PKCs act as a “fulcrum” that is able to up- or down-regulate the signaling pathway, resulting in cell proliferation or apoptosis. In this section, the most intriguing evidence about the relative contribution of each PKC isoenzyme to cell survival and death pathway is summarized.

PKCα has emerged as an important isoform in promoting cell survival. In several cell lines, including endothelial cells [99] and glioma cells [100,101], apoptosis was induced as a result of cellular PKCα depletion. Although the mechanism by which PKCα prevents apoptosis is only partially known, one target that has been identified is the anti-apoptotic Bcl-2 protein. In HL-60 cells, PKCα colocalized with Bcl-2 in the mitochondria [102] while other experiments, with murine growth factor-dependent cell lines, demonstrated that PKCα phosphorylated Bcl-2 at Ser70 [103]. Phosphorylation of this site had the effect of stabilizing Bcl-2 and enhancing its ability to prevent apoptosis. Another possible target for PKCα is Raf-1, which has been shown to mediate the anti-apoptotic function of PKB/Akt in hematopoietic cells through a PKC-dependent mechanism [104]. From this prospective, in various types of cancer cells, this anti-apoptotic role of PKCα resulted in an increase in cell proliferation [105] and survival [106]. In this regard, it has been demonstrated that the antisense of PKCα inhibits cell proliferation *in vitro* and tumorigenicity *in vivo* in nude mice xenografts of human glioblastoma and lung cancer cells [107,108]. In addition, microinjection of antibodies against PKCα also inhibits cell growth and differentiation of neuroblastoma cells [109]. Moreover, PKCα knockdown impaired tumor growth and reduced the activation of Akt and ERK, suggesting that PKCα is an upstream regulator of these critical growth and survival signaling pathways [110]. Although the majority of published work suggests a suppressive role for PKCα in apoptosis, conflicting data, indicating a pro-apoptotic function, has been observed. In human prostate cancer cell lines, the presence of PKCα in the mitochondrial membrane was associated with apoptosis while its absence corresponded to resistance to cell death [111]. In addition, PKCα was shown to mediate the activation of caspase-3 in renal proximal tubule cells treated with cisplatin [112]. Furthermore, the stable overexpression of PKCα in LNCaP cells, a widely used cellular model of androgen-dependent prostate cancer, suggested that the activation of this isoenzyme was critical to the PMA apoptotic response [111]. This particular study demonstrated a strong correlation between the presence or absence of PKCα in the membrane and the apoptosis induction or resistance, respectively. Moreover, it has been reported that PKCα can also exert growth inhibitory functions in intestinal, pancreatic and mammary cells [113-115]. In the case of intestinal cells, PMA treatment causes cell-cycle arrest in G1 in a PKCα-dependent manner. G1 arrest occurs with a corresponding increase in the expression levels of the cyclin-dependent kinase inhibitors p21 and p27, a decrease in retinoblastoma (RB) phosphorylation and a sustained ERK activation [115,116]. Recent studies carried out by using the PKCα null mouse [47] and the phenotypical analysis of PKCα knockouts in colorectal cancer [36] have suggested that PKCα has a role in tumor suppressor. On the basis of these findings, it is clear that PKCα, in a tumor-specific manner, assumes different roles in the control of cell survival and death, but not only, since PKCα also plays a critical role in the induction of chemosensitivity. Specifically, it confers resistance to drugs like cisplatin in prostate cancer cells [117], etoposide in leukemia cells [103] and tamoxifen in breast cancer cells [118].

PKCβI and PKCβII derive from a single gene by alternative splicing and are differentially involved in cell growth and apoptosis [119,120]. Initial studies have demonstrated that PKC-βII promotes cellular proliferation in human leukemia cells and colon cancer cell lines [121,122] and a positive effect of PKC-βI on the growth and proliferation of neuroblastoma cells has been found [123]. Subsequent studies have shown that the expression of PKCβII in the colon of transgenic mice leads to hyperproliferation and increased susceptibility to colon carcinogenesis [57,124], whereas PKCβI seems to act as a survival mediator in response to chemotherapeutic agent-induced apoptosis in gastric cancer [125,126]. In addition, the expression of the oncogene v-*abl* causes translocation of PKCβII to the nucleus, thereby preventing apoptosis and confirming that PKCβII is anti-apoptotic [127]. Moreover, a mitotic lamin kinase has been identified as a target for PKCβII, and its interaction with this substrate promotes cell survival and proliferation [128,129]. In fact, lamin B is phosphorylated by the PKC βII after treatment with bryostatin [130], an activator of PKC, and this phosphorylation leads to the solubilization of lamin B.

Possible pro-apoptotic activity of PKCβ has also been reported. Activation of PKCβI by 12-deoxyphorbol 13-phenylacetate 20-acetate (DOPPA), which is a selective activator of this isoform *in vitro*, induced apoptosis in HL60 cells [131] indicating that PKCβI and PKCβII might have opposite roles in the regulation of apoptosis. In a subsequent study, PKCβ was demonstrated as being necessary in the targeting of stress-activated protein kinase (SAPK) to the mitochondria [132]. SAPK was shown to, not only interact with, but also phosphorylate the anti-apoptotic Bcl-2 family member Bcl-x(L) in the mitochondria, resulting in promoting the release of cytochrome c.

The activation of PKCδ is associated with the inhibition of cell cycle progression and its downregulation is linked to tumor promotion, suggesting that PKCδ may have a negative effect on cell survival [70,133]. In many cases, the growth-inhibitory effects of PKCδ have been linked to changes in the expression of factors that influence cell cycle progression. Furthermore, we know that PKCδ plays an essential role in the genotoxic stress response leading to apoptotic cell death in many cell types. In fact, PKCδ is activated by numerous apoptotic stimuli, including genotoxins [67], oxidative stress [68,69,134] and death receptors [135]. Conversely, the inhibition of PKCδ with rottlerin or the expression of PKCδKD (kinase dead PKCδ) inhibits apoptosis induced by a variety of stimuli [136]. Depending on the cell types and apoptotic stimuli, PKCδ translocates to nearly all subcellular organelles, including the nucleus [137], mitochondria [138], Golgi complex, endoplasmic reticulum and plasma membrane [139]. At each subcellular organelle, PKCδ phosphorylates different substrates leading to cell death. Whilst the identification of these substrates is critical in order to understand the mechanism of PKCδ, it has been very challenging to identify physiologic substrates in each organelle. Furthermore, although putative PKCδ substrates have been identified in apoptotic cells, the molecular mechanisms through which PKCδ regulates apoptosis are not known. Nuclear proteins comprise the largest group of PKCδ substrates identified in apoptotic cells. These include lamin B, the checkpoint protein hRad9 and DNA protein kinase, all of which have been shown to be phosphorylated by PKCδ in genotoxin-treated cells [140,141]. PKCδ may also regulate the transcription of death genes through activation or inactivation of transcription factors such as p53, p73 and STAT1 [142,143]. In this regard, it has been demonstrated that TP53 functions as a novel nuclear effector of PKCδ-mediated apoptosis [144]. Specifically, PKCδ activates transcription factor Btf to bind with the TP53 promoter. Moreover, the disruption of Btf-mediated TP53 gene transcription leads to the suppression of TP53-mediated apoptosis following genotoxic stress [145]. Interestingly, it has been demonstrated that PKCδ regulates p53 not only that at transcriptional level, but also at post-translational levels. For example, in smooth muscle cells apoptosis is triggered by a pathway that involves PKCδ, the intermediary p38 MAPK, and the downstream target tumor suppressor p53 [146]. In another study, carried out on dopaminergic neurons, it has been shown that nitration-mediated activation of PKCδ induces the phosphorylation of p53 at the Ser15 residue, which increases its protein stability, thereby contributing to the nitric oxide-mediated apoptosis [147]. PKCδ also activates the JNK pathway through phosphorylation and activation of MEKK1 (MEK kinase 1) [148]. In addition, PKCδ has been reported to interact with c-Abl in response to both genotoxic and oxidative stress [149]. Significantly, other studies have identified reciprocal regulation of PKCδ by NF-κB by showing that a NF-κB-responsive regulatory element in the PKCδ promoter links TNFα stimulation to an increase in PKCδ mRNA and protein expression [150].

However, the ability of PKCδ to activate an apoptotic program is regulated by three key steps. Firstly, the transduction of a “death” signal to PKCδ by a DNA damage sensor pathway that may occur via phosphorylation of PKCδ at specific residues [151]. Secondly, the transitory accumulation of the activated PKCδ in the nucleus where it is cleaved by caspase 3 [142], and finally, the nuclear accumulation of PKCδ resulting in the cells undergoing apoptosis [137]. In regard to these events, it has recently been found that PKCδ contains a nuclear localization sequence that is required for its nuclear import. Moreover, it has been proposed that PKCδ full-length (FL) may act as an apoptotic sensor, since its nuclear accumulation precedes the activation of any of the known components of the apoptotic pathway in etoposide-treated parC5 cells [136]. In the absence of an apoptotic signal, PKCδ is retained in the cytosol while apoptotic signals, such as etoposide, induce post-translational modifications in the PKCδ which may allow its nuclear accumulation [152]. Active caspase 3 also accumulates in the nucleus in response to etoposide, resulting in the cleavage of PKCδ and generation of the δ catalytic-fragment (CF). In contrast with PKCδFL, δCF is constitutively present in the nucleus, where it presumably regulates apoptosis through the phosphorylation of proteins involved in cell damage, as well as other apoptotic mediators. On the basis of these findings, it is possible to suggest that a strict regulation of nuclear import and export of PKCδ is critical for cell survival and that caspase cleavage of PKCδ in the nucleus signals an irreversible commitment to apoptosis [152].

In addition to its apoptotic functions, PKCδ has also been reported to exert antiapoptotic effects. Thus, PKCδ protects macrophages from apoptosis induced by nitric oxide [153] and exerts antiapoptotic effects on glioma cells treated with TRAIL [154].

Similarly, PKCδ promotes survival and chemotherapeutic drug resistance of non–small cell lung cancer cells [155]. As previously described in this section, one of the factors that may contribute to the diverse effects of PKCδ on cell fate is its different subcellular localizations. In fact, on one hand, the translocation of PKCδ to the Golgi, mitochondria and nucleus has been associated with proapoptotic effects [155]. On the other hand, its translocation to the endoplasmic reticulum (ER) results in antiapoptotic effects [154]. The role of PKCδ in the ER and the mechanisms involved in its antiapoptotic effects are currently not fully understood. However, there are several apoptosis-related proteins which reside in the ER and play an important role in the regulation of cell survival. One possible PKCδ substrate in the ER is Bcl2, which regulates the “cross-talk” between the ER and the mitochondria during cell apoptosis [156]. Moreover, the phosphorylation of AKT [154] and HSP25 [157] is associated with the antiapoptotic effects of PKCδ. Finally, a novel PKCδ isoenzyme, PKCδVIII, has been recently identified in human teratocarcinoma (NT2) cells [158]. In both *in vivo* and *in vitro* assays, PKCδVIII has been demonstrated to be resistant to caspase-3 cleavage. In addition, the overexpression or down-regulation of the PKCδVIII isoenzyme suggests its antiapoptotic function. On the basis of this information, it is possible to assume that PKCδ-dependent signaling not only represents a mechanism for protecting cells from stress conditions and a mechanism for promoting apoptosis to eliminate irreversibly damaged cells, but also provides a mechanism for switching or regulating cells between survival and death.

Involvement of PKCε in the apoptotic pathways has been disclosed in cancer research [159,160] and supported by the finding that PKCε knockout mice exhibited significantly decreased survival [161]. Several studies demonstrated that PKCε plays a protective role during receptor-mediated cell death and it has been reported that cellular susceptibility to TRAIL correlates with PKCε level [162]. In fact, introduction of dominant-negative PKCε [163] or knockdown of PKCε [154] sensitized glioma cells to apoptosis. Moreover, PKCε not only regulates apoptosis but it is also cleaved by caspases in response to several apoptotic stimuli, including chemotherapeutic agents, starvation and TNF [160]. Contradictory results have been obtained about the role of caspase-mediated PKCε cleavage and apoptosis, suggesting that the cellular context may play an important role in deciding whether proteolytic cleavage of PKCε will induce, inhibit or have no effect on apoptosis.

From several studies it appears clear that the antiapoptotic effects of PKCε were mediated by an increase in Akt phosphorylation and activity [164]. In this regard, the interaction of Akt and PKCε was associated with an increase in Akt phosphorylation at Ser473 and consequently, resistance to apoptosis. Interestingly, signaling via both proteins was required for efficient MAPK activation, suggesting that the PKCε–Akt complex can cross-talk with a third pathway to mediate its antiapoptotic effects [165]. PKCε may also enhance Akt activity indirectly, through a positive feed-back loop comprising also integrins [164].

Contrary to the data cited above, it has been also reported that PKCε negatively regulates Akt function and this was associated with increased apoptosis [166]. This inhibitory effect was associated with a decrease in Akt phosphorylation.

Additional studies have attributed also a regulatory role on Bcl-2 family members to PKCε. In fact, it has been shown that PKCε enhances antiapoptotic Bcl-2 members while it inhibits the proapoptotic members of this family [162]. Moreover, it has been reported that the development of pregnancy-dependent mammary tumors to malignant tumors was accompanied by an intense expression of Bcl-2 and was associated with the overexpression of PKCε [167] In addition, it has recently been found that overexpression of PKCε in MCF-7 cells increased Bcl-2 mRNA and protein level and, concomitantly, decreased the proapoptotic protein Bid. This dual regulation of pro- and antiapoptotic members of the Bcl-2 family contributed to TRAIL resistance. Moreover, it has been reported that PKCε-deficient cells were sensitive to PMA-induced apoptosis and the overexpression of PKCε in these cells conferred resistance to PMA-mediated apoptosis by preventing Bax activation and translocation to mitochondria [168].

Higher levels of PKCε, in small cell lung cancer (SCLC), were associated with higher Bcl-XL and X-linked inhibitor of apoptosis (XIAP) protein levels [169]. Moreover, a high percentage of patients with SCLC die in consequence of the chemoresistance that may be due to the increased expression of some antiapoptotic proteins [170]. The strict link between PKCε and chemoresistance has recently been demonstrated by a study of Bourgulgon *et al.* In particular, this study indicate that the hyaluronan (HA)-induced interaction between CD44 (a primary HA receptor) and PKCε increases the phosphorylation of the stem cell marker, Nanog. Moreover, HA-CD44-mediated PKCε-Nanog signaling mediates miR-21 production, which in turn, exerts its influence on tumor cell-specific functions, including anti-apoptosis and chemoresistance. This newly discovered PKCε-Nanog signaling pathway should provide important drug targets for sensitizing tumor cell to apoptosis and overcoming chemoresistance in HA-CD44-activated breast cancer cells [171].

## 4. PKC Modulators: from the Laboratory to Its Clinical Employment

The participation of PKC isoenzymes in cancer, either by antagonizing or promoting malignant growth, supports the notion that PKCs could be potential direct targets for anticancer therapy. In fact, several PKC modulators are currently in clinical trials as chemotherapeutic agents.

PKC inhibitor therapy is currently employed in human clinical trials, both alone and in combination with other modalities [55,172]. Different strategies have been devised in the drug development of PKC inhibitors and these include ATP and protein substrate binding pocket inhibitors, small molecule kinase inhibitors, biologic modulators of PKC and anti-sense oligonucleotides.

*Staurosporine*, the first reported ATP competitive PKC inhibitor [173], is produced by *streptomymes Sp* and shows an anti-proliferative action. Although this compound lacks specificity for PKC isoforms, it has served as a lead compound from which many other PKC inhibitors have been developed, among them, Midostaurin and Enzastaurin, which have been employed in anti-cancer clinical trials [174].

*Midostaurin (PKC412 or n-benzoylstaurosporine*), similar to UCN01 (7-hydroxystaurosporine), was the first PKC inhibitor to have been evaluated in oncology clinical trials [175].

This compound exhibits selectivity for the ATP binding sites, but shows modest isoenzyme specificity. In pre-clinical studies, midostaurin has shown a broad range of anti-tumor activities, synergizing with conventional cytotoxic agents [176,177]. From *in vitro* and *in vivo* studies, encouraging results have been obtained, in particular, it has been demonstrated that midostaurin inhibits PKC activity in melanoma cells [178] and delays the development of lung metastasis in mice [179]. Moreover, this compound has displayed some clinical activity as a single agent and was able to potentiate the anti-tumor activity of some of the clinically-used cytotoxins (Taxol® and doxorubicin) [180,181]. Midostaurin was shown to have biological activity in low grade lymphoproliferative disorders like B-chronic lymphocytic leukemia [182,183] and acute myeloid leukemia [184,185]. Midostaurin was well-tolerated in a phase I study, with the main toxicities being nausea, vomiting, diarrhea and fatigue. Therefore, a phase II trial was investigated in patients with malignant melanoma and some of them, with accessible tumors, were biopsied to examine drug efficacy. Unfortunately, in these latter studies, midostaurin failed to statistically demonstrate significant clinical activity [186].

*Enzastaurin (LY317615)* is an oral serine/threonine kinase inhibitor that was originally evaluated in human tumor xenograft-bearing mice for its antiangiogenic activity [187]. At low concentrations, enzostaurin inhibits PKCβ but, at higher concentrations, it acts unspecifically, inhibiting the other PKC isoenzymes [188].

Moreover, the anti-tumor effects of enzastaurin are mediated through interference with the phosphatidylinositol3-kinase (PI3K)/Akt pathway [60,189-191]. Several studies have shown that enzastaurin exhibits direct growth inhibiting effects on a wide array of cultured human tumor cells [60,189-193] and in animal models, it showed anti-tumor and anti-angiogenic activity in various malignancies [194]. Currently, enzastaurin is being evaluated in several clinical trials and it appears to be well-tolerated at doses from 20 to 750 mg/day in patients with advanced solid tumors [195] and the recommended oral daily dose was 525 mg [196]. Although this was a phase I study, several patients with lung cancer, colorectal carcinoma and renal carcinoma demonstrated prolonged disease stabilization [195].

The PKCβ and PI3K–Akt pathways are frequently activated in glioblastoma, making this an attractive tumor type in which to further investigate enzastaurin. Reports from a phase II trial in patients with recurrent high-grade gliomas were promising [197]. A phase III study showed that treatment with enzastaurin was well-tolerated and associated with prolonged freedom from progression in a small subset of patients with relapsed or refractory diffuse large B-cell lymphoma (DLBCL) [198]. Moreover, a large global phase III trial of standard induction therapy (prednisone/ rituximab), with or without enzastaurin consolidation, has recently been initiated in patients with newly diagnosed, high-intermediate/high-risk DLBCL [199]. Finally, combination studies of enzastaurin with gemcitabine and cisplatin have been investigated and preliminary reports from this phase I study, look promising [200].

In conclusion, enzastaurin is a very promising anticancer agent *per se* and it is a good candidate for different combination regimens with other novel targeted agents and cytotoxic drugs commonly used in the clinical setting.

*UCN-01 (7-hydroxystaurosporine)*, a staurosporine analogue isolated from the culture broth of *Streptomyces* species [201], is an inhibitor of cPKC and nPKC isoenzymes [202] and also of cdk1 and cdk2 [203-205]. Pre-clinical models have demonstrated synergistic activity of UCN-01 with a number of cytotoxic agents [206-208]. For this reason, several phase I studies have been conducted with UCN-01 as a single agent and in combination with cytotoxic chemotherapy [209-211]. In the single-agent phase I study, pharmacokinetic data revealed that UCN-01 has a very small volume of distribution, low systemic clearance and a prolonged half-life of elimination (>200 h) [212]. Three phase I combination studies, in which UCN-01 was combined with cisplatin [211], 5-fluorouracil [210] and topotecan [213] involving patients with solid tumors have been carried out. Moreover, this compound is currently being employed in clinical trials for leukemia, non-small cell lung cancer (NSCLC), and lymphoma.

*Bryostatins* are a family of at least 20 macrocyclic lactones derived from the marine bryozoan *Bulgula neritina* [214]. Bryostatin is an activator of DAG/phorbol ester sensitive PKC isoforms and induces differential downregulation of isoforms in cells causing suppression of selective responses [215,216].

The prototype compound for this class of drugs is bryostatin 1.

*Bryostatin 1* is a potent modulator of PKC activation [214,216,217]. In particular, short-term exposure with bryostatin 1 results in cPKC and nPKC activation and translocation to the nuclear membrane [218]. Conversely, prolonged exposure with bryostatin 1 results in membrane depletion of PKC and decreased PKC activity [219]. Due to bryostatin 1 showing significant growth-inhibitory activities against various cancer cell lines, its clinical application has been examined in phase I and II studies using a wide range of tumor types [220-223]. In phase I trials, bryostatin 1 showed anti-tumor activity, but phase II studies using bryostatin 1 alone were disappointing in melanoma [224], colorectal cancer [225] and gastric carcinoma [226]. The dose-limiting toxicity (DLT) in all studies was myalgia and localized phlebitis at the infusion site. Significant increases in plasma concentrations of TNFα and IL6, chosen as markers of PKC inhibitory activity, were observed when 50 μg per m^2^ of bryostatin 1 was given as a weekly one hour infusion for three weeks out of four [223]. As the application of paclitaxel followed by bryostatin 1 significantly reduced tumor growth in mice [227], phase II studies of bryostatin 1, in combination with other cytotoxic agents, were tried in pancreatic and prostate cancer, and renal cell and gastric carcinoma [39,220,226]. In particular, an enhanced response to paclitaxel by bryostatin 1 was observed in advanced gastric or gastroesophageal junction adenocarcinoma [226] and in advanced esophageal and gastroesophageal junction cancer [228]. Recently, other combination (bryostatin 1 and vincristine) phase II study had efficacy in patients with aggressive B-cell non-Hodgkin lymphoma [229]. Moreover, these studies have emphasized the importance of the schedule sequence, the administration of bryostatin 1 before cisplatin, vincristine and gemcitabine being synergistic, while synergy with paclitaxel required the administration of bryostatin 1 after paclitaxel [227]. In addition, there is substantial evidence that bryostatin 1 is a potent immunostimulant [230,231] suggesting that a single target of bryostatin 1 is not likely. In fact, an upregulation of IL2 by PKC has been reported and a phase II study was conducted combining IL2 with bryostatin 1 in patients with renal cell carcinoma. Although it was well tolerated, the addition of bryostatin 1 did not appear to improve response rates and there was no significant effect on T-cell expansion, activation or cytokine production [232]. Given that bryostatin has pleiotropic effects, it is not clear which are the most promising targets to measure in terms of predicting anticancer activity in any given tumor type. Currently there is no data on the predictive value of individual PKC isoenzymes in terms of bryostatin efficacy.

*Ingenol-3-angelate (PEP005)* is a novel compound extracted and purified from *Euphorbia peplus*. Chemically, PEP005 is structurally analogous to phorbol esters and is a potent modulator of PKC isoenzymes [233]. PEP005 was shown to modulate PKCs by activating PKCδ in human myeloid leukemia cancer cell lines, thereby inducing cellular apoptosis in melanoma [234] and in colon cancer models [235] through the inhibition of the AKT signaling pathway [236]. The antiproliferative effects of PEP005 were related to cell cycle inhibition in the G1 phase as well as the induction of apoptosis. Considering that concentrations required to observe cytotoxic effects, apoptosis, and/or cell cycle blockage may be limited by toxicity when PEP005 is given to patients with solid tumors, it is essential to evaluate combinations which could allow the use of lower concentrations of PEP005 and which might improve the cytotoxic effects of already used anticancer agents. This compound is currently in phase III clinical trials for the treatment of actinic keratosis [237] and phase II for non-melanoma skin cancer [238]. PEP005 exposure induced necrosis of tumor cells and caused a local moderate acute inflammatory response, which resolved over 5–10 days, leaving a favorable cosmetic effect [239]. It has been observed [235] that the action that PEP (4–1000 ng/mL) has on PKC is important for stimulating the observed inflammatory response [240], whereas, PKC activation is not required for inducing primary necrosis [239].

In addition, PEP005 emerges as a novel immunostimulatory chemotherapeutic agent that not only ablates the treated tumor, but in doing so also generates anti-cancer CD8 T cells that can synergize with CD8 T cell-based immunotherapies to regress distant secondary tumors [241]. However, only a limited number of reports of combination therapies have demonstrated that treatment of one tumor can lead to regression of distant pre-existing (secondary) tumors [242,243].

*Curcumin* is a natural polyphenol derived from the plant *Curcuma longa*, commonly called turmeric. This compound is a potent inhibitor of PKC [244] and acts by competing with calcium for the binding domain [245]. A number of preclinical studies showed that curcumin exhibits anti-tumor effects against a wide variety of cancers [244,246-250]. Several phase I and phase II clinical trials indicate that curcumin is quite safe and may exhibit therapeutic efficacy.

In particular, it has been demonstrated that a standardized formulation of curcuma extract could be efficacy in patients with advanced colorectal cancer [251]. Moreover, a study conducted in patients with familial adenomatous polyposis showed that curcumin could have a potential role in inhibiting this malignancy [252]. In addition, in a phase I clinical trial, a daily curcumin dose of 8000 mg taken for three months resulted in histological improvement of precancerous lesions in patients having uterine cervical intraepithelial neoplasm, intestinal metaplasia, bladder cancer and oral leucoplakia [253].

Finally, clinical trials have demonstrated the efficacy of curcumin in patients with pancreatic cancer [254] and prostatic neoplasia [255].

*Aprinocarsen (ISIS 3521)* is an antisense oligonucleotide that induces a concentration-dependent reduction of PKCα protein levels [256]. Continuous infusion of aprinocarsen was associated with greater uptake into tissues, prolonged inhibition of PKCα mRNA and reduced plasma concentrations. In phase I studies the main toxicities were fatigue, nausea, vomiting, fever and chills, and thrombocytopenia. Anti-tumor activity was shown in non-Hodgkin lymphoma and ovarian carcinoma in a phase I study [257], but no clinical benefit was observed in a phase II study in patients with recurrent high-grade astrocytomas [108] or with breast cancer. Phase I and II studies of aprinocarsen in combination with carboplatin and paclitaxel in NSCLC achieved a 42% response rate, suggesting potentiation of chemotherapy activity [258,259].

However, two randomized phase III studies in NSCLC failed to show a benefit from the addition of aprinocarsen to gemcitabine and cisplatin or to paclitaxel and carboplatin [260].

*Nucleoside analogs*, among them *ARC* (NSC 188491, SMA-491), 4-amino-6-hydrazino-7-β-d-ribofuranosyl-7H-pyrrolo-(2,3-d)-pyrimidine-5-carboxamide and *sangivamycin* show *in vitro* a marked anti-cancer activity. This class of drug affects quiescent and proliferating cells by impacting DNA and RNA synthesis. Moreover, these two compounds are able to inhibit positive transcription elongation factor b (pTEFb), PKC and VEGF secretion [261]. The identical behavior of ARC and sangivamycin is interesting given that several reports exist of Phase I trials of sangivamycin in patients with a range of malignancies [262,263].

*Perifosine octadecyl-(1,1-dimethyl-4-piperidylio) phosphate* is a lipophilic orally bioavailable synthetic acetylphospholipid. It has shown antitumor activity in preclinical models. Although the exact mechanism of action is not yet fully understood, perifosine interacts with cell membranes and inhibits regulatory signal proteins including PKC [264]. This compound was the object of a phase I study and partial positive results were obtained in patients with chondrosarcoma and uterine sarcoma [265].

*Disulfiram*, Bis(N,N-(diethylthiocarbamoyl) disulfide (DSF), is an FDA-approved drug [266]. Its anticancer activity has been associated with S-thiolation and regulatory modulation of PKC isoenzymes [267]. Recently, it has been demonstrated that the redox active copper(II)-bis-N,N-diethyl-dithiocarbamate-derivative DSF was the causative agent underlying DSF-induced cancer cell apoptosis [266]. Recently, the potential role of DSF as a redox chemotherapeutic agent in metastatic melanoma has been reviewed [268,269]. The safety profile and prior clinical experience with DSF have encouraged ongoing clinical phase I and phase II studies in human metastatic melanoma (Clinical-Trials.gov Identifier: NCT00256230). A potential prooxidant potentiation that results in improved therapeutic benefit may exist between DSF and arsenic trioxide, a combination currently evaluated in patients with metastatic melanoma who underwent at least one prior systemic therapy (ClinicalTrials.gov Identifier: NCT00571116). Initial assessment of the effect of the addition of disulfiram to standard chemotherapy in NSCLC is the subject of an ongoing phase I trial (ClinicalTrials.gov Identifier: NCT00312819). Moreover, another phase I study examines disulfiram and copper gluconate for the treatment of refractory solid tumors involving the liver (ClinicalTrials.gov Identifier: NCT00742911).

## 5. Conclusions

**Figure 2.**
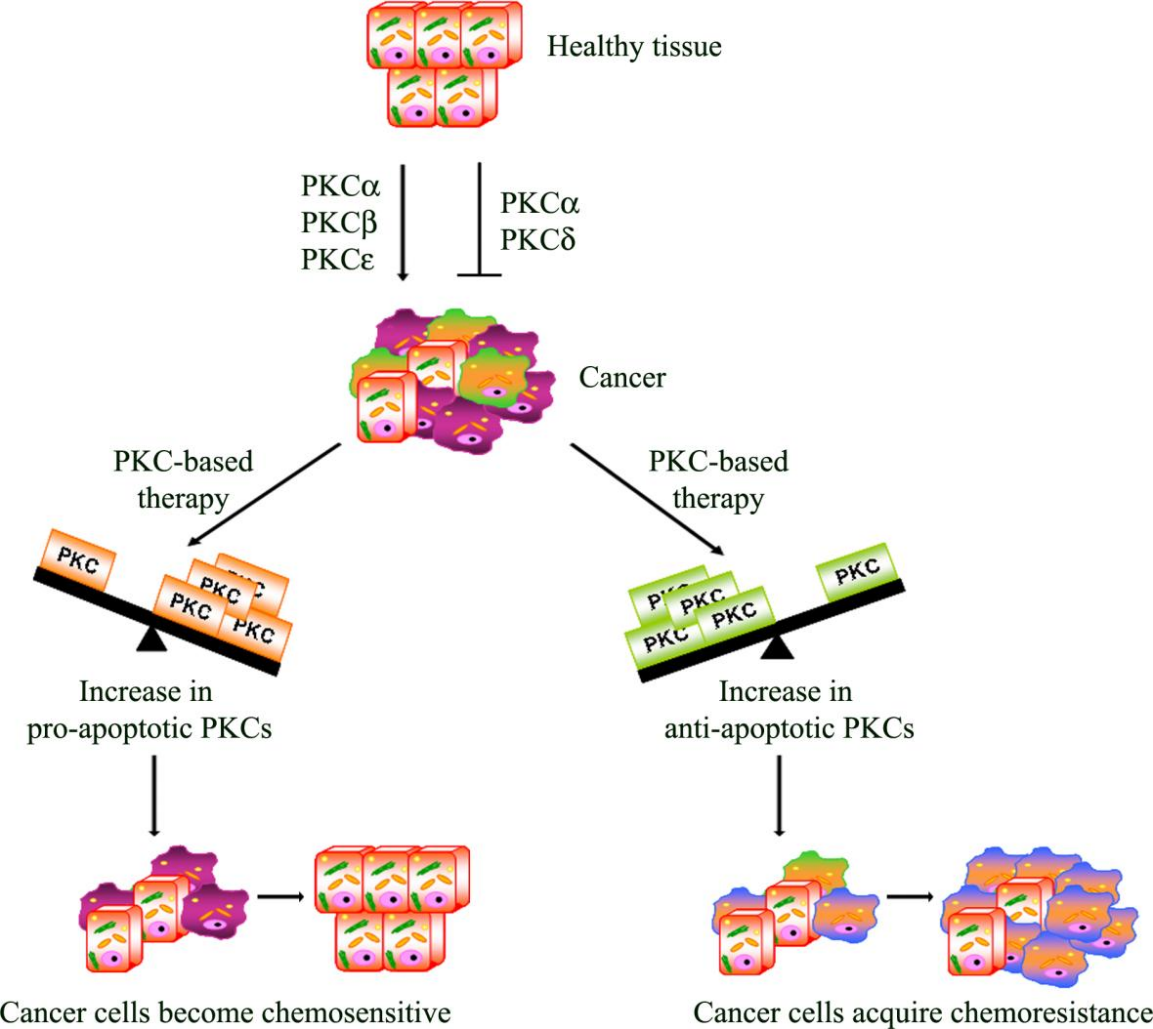
Schematic representation of the role of PKCs in cancer development and implication of their modulation in clinical therapy. PKC isoforms may act as tumor promoters or as tumor suppressors. Moreover, the activation of PKCs can be associated with resistance (increase in anti-apoptotic PKCs) but can also increase sensitivity (increase in pro-apoptotic PKCs) to chemotherapy.

PKC-dependent pathways participate in the resistance to chemotherapeutic treatments through the modulation of multi-drug transporters [98] and/or the regulation of apoptosis. Many studies have focused on rendering the chemotherapy more effective in order to overcome the evasion from apoptosis. In this regard, as we have reported, PKC isoform activation can be associated with chemoresistance but can also increase the sensitivity to chemotherapy (Figure 2). Moreover, emerging evidence also suggests that dysregulation of PKC isoenzymes is commonly observed in several malignancies and has been associated with promotion and propagation of cancer. For these reasons, PKC isoforms are attractive targets to kill cancer cells and increase the efficacy of chemotherapy. Natural compounds, small molecules and genetic approaches have been developed against PKCs, but the interpretation of clinical trials evaluating these approaches has been confusing and limited. The current PKC inhibitors clinically employed are relatively non-specific in their actions and, given the complexity of the functions and interactions of PKC isoenzymes, it is perhaps not surprising that agents targeting multiple isoenzymes give mixed results. Moreover, evidence from cell cultures and the early phases of clinical trials suggests promising results for the combination of conventional cytotoxic drugs with the current PKC inhibitors (Table 1). However, it is necessary to underly that the optimal combination and the sequence in which these drugs can be used needs to be carefully evaluated, bearing in mind that the efficacy of this strategy might be tumor type-dependent. Furthermore, additional translational research is needed to demonstrate if the modulation of “upstream–downstream” targets of PKC-dependent pathway might be more effective than either agent alone and if this approach will be beneficial in altering tumor progression. However, the therapeutic limitations of current drugs and the encouraging results of preclinical and clinical studies justify the continued search for drugs aimed at triggering the apoptotic response.

## Figures and Tables

**Table 1.** PKCs modulators and their clinical employment in human cancer.

**PKCs modulator**	**Tumor type**	**Ref.**
Midostaurin	B-chronic lymphocytic leukemia	[182,183]
	Acute myeloid leukemia	[184,185]
	Malignant melanoma	[186]
Enzastaurin	Lung cancer	[195]
	Colorectal carcinoma	[195]
	Renal carcinoma	[195]
	High-grade gliomas	[197]
	Diffuse large B-cell lymphoma	[198,199]
UCN-01	Leukemia	Under study
	Non-small cell lung cancer	Under study
	Lymphoma	Under study
Bryostatin 1	Gastric carcinoma	[220]
	Adenocarcinoma	[226]
	Esophageal and gastroesophageal cancer	[228]
	Aggressive B-cell non-Hodgkin lymphoma	[229]
Ingenol-3-angelate	Actinic keratosis	[237]
	Non-melanoma skin cancer	[238]
Curcumin	Advanced colorectal cancer	[251]
	Familial adenomatous polyposis	[252]
	Uterine cervical neoplasm	[253]
	Intestinal metaplasia	[253]
	Bladder cancer	[253]
	Oral leukoplakia	[253]
Aprinocarsen	Non-Hodgkin lymphoma	[257]
	Ovarian carcinoma	[257]
Perifosine octadecyl	Chondrosarcoma	[265]
phosphate	Uterine sarcoma	[265]
Disulfiram	Metastatic melanoma	[268,269]
